# Development and Implementation of the Ebola Traveler Monitoring Program and Clinical Outcomes of Monitored Travelers during October – May 2015, Minnesota

**DOI:** 10.1371/journal.pone.0166797

**Published:** 2016-12-01

**Authors:** Aaron DeVries, Pamela Talley, Kristin Sweet, Susan Kline, Patricia Stinchfield, Pritish Tosh, Richard Danila

**Affiliations:** 1 Infectious Disease Section, Minneapolis VA Medical Center, Minneapolis, Minnesota, United States of America; 2 School of Medicine and Public Health, University of Minnesota, Minneapolis, Minnesota, United States of America; 3 Epidemic Intelligence Service, Division of Science Education and Professional Development, Centers for Disease Control and Prevention, Atlanta, Georgia, United States of America; 4 Infectious Disease Epidemiology, Prevention and Control Division, Minnesota Department of Health, St. Paul, Minnesota, United States of America; 5 Infectious Disease Division, University of Minnesota Medical School, Minneapolis, Minnesota, United States of America; 6 Children's Hospitals and Clinics of Minnesota, St. Paul, Minnesota, United States of America; 7 Division of Infectious Disease, Mayo Clinic, Rochester, Minnesota, United States of America; RIVM, NETHERLANDS

## Abstract

**Background:**

In October 2014, the United States began actively monitoring all persons who had traveled from Guinea, Liberia, and Sierra Leone in the previous 21 days. State public health departments were responsible for monitoring all travelers; Minnesota has the largest Liberian population in the United States. The MDH Ebola Clinical Team (ECT) was established to assess travelers with symptoms of concern for Ebola virus disease (EVD), coordinate access to healthcare at appropriate facilities including Ebola Assessment and Treatment Units (EATU), and provide guidance to clinicians.

**Methods:**

Minnesota Department of Health (MDH) began receiving traveler information collected by U.S. Customs and Border Control and Centers for Disease Control and Prevention staff on October 21, 2014 via encrypted electronic communication. All travelers returning from Liberia, Sierra Leone, and Guinea during 10/21/14–5/15/15 were monitored by MDH staff in the manner recommended by CDC based on the traveler’s risk categorization as “low (but not zero)”, “some” and “high” risk. When a traveler reported symptoms or a temperature ≥100.4° F at any time during their 21-day monitoring period, an ECT member would speak to the traveler and perform a clinical assessment by telephone or via video-chat. Based on the assessment the ECT member would recommend 1) continued clinical monitoring while at home with frequent telephone follow-up by the ECT member, 2) outpatient clinical evaluation at an outpatient site agreed upon by all parties, or 3) inpatient clinical evaluation at one of four Minnesota EATUs. ECT members assessed and approved testing for Ebola virus infection at MDH. Traveler data, calls to the ECT and clinical outcomes were logged on a secure server at MDH.

**Results:**

During 10/21/14–5/15/15, a total of 783 travelers were monitored; 729 (93%) traveled from Liberia, 30 (4%) Sierra Leone, and 24 (3%) Guinea. The median number monitored per week was 59 (range 45–143). The median age was 35 years; 136 (17%) were aged <18 years. Thirteen of 256 women of reproductive age (5%) were pregnant. The country of passport issuance was known for 720 of the travelers. The majority of monitored travelers (478 [66%]) used a non-U.S. passport including 442 (61%) Liberian nationals. A total of 772 (99%) travelers were “low (but not zero)” risk; 11 (1%) were “some” risk. Among monitored travelers, 43 (5%) experienced illness symptoms; 29 (67%) had a symptom consistent with EVD. Two were tested for Ebola virus disease and had negative results. Most frequently reported symptoms were fever (20/43, 47%) and abdominal pain (12/43, 28%). During evaluation, 16 (37%) of 43 travelers reported their symptoms began prior to travel; chronic health conditions in 24 travelers including tumors/cancer, pregnancy, and orthopedic conditions were most common. Infectious causes in 19 travelers included upper respiratory infection, malaria, and gastrointestinal infections.

**Discussion:**

Prior to 2014, no similar active monitoring program for travelers had been performed in Minnesota; assessment and management of symptomatic travelers was a new activity for MDH. Ensuring safe entrance into healthcare was particularly challenging for children, and pregnant women, as well as those without an established connection to healthcare. Unnecessary inpatient evaluations were successfully avoided by close clinical follow-up by phone. Before similar monitoring programs are considered in the future, careful thought must be given to necessary resources and the impact on affected populations, public health, and the healthcare system.

## Introduction

The Ebola virus disease (EVD) epidemic likely began in Guinea late in 2013 and rapidly spread to Sierra Leone and Liberia [1, 2, 3]. In October 2014, out of concern for possible importation of EVD into the United States, the U.S. Centers for Disease Control and Prevention (CDC) and other federal agencies began a coordinated program of active monitoring for persons who had recently traveled from a country where EVD was widespread [4]. All travelers from Ebola-affected countries had their flight itineraries altered such that they were required to arrive through one of five U.S. ports of entry: 1) Dulles International Airport, 2) John F. Kennedy International Airport, 3) Newark Liberty International Airport, 4) Hartsfield-Jackson Atlanta International Airport, or 5) O’Hare International Airport [5]. Prior to departure from affected countries, United States government staff at airports within affected countries screen travelers for recent exposure to EVD, take temperatures, and assess for symptoms [4, 6]. Individuals were not allowed to board planes if they were exhibiting fever or other symptoms or until 21 days had passed since their last EVD exposure. Upon arrival at one of the five U.S. airports, travelers were screened again for EVD exposures in the 21 days prior to their arrival in the United States. Screening included an assessment of current symptoms, measuring temperatures, and collecting specific final destination information, including planned flights to other U.S. destinations Travelers were given information on the symptoms and transmission of EVD, a thermometer and a diary with instructions to take and record their temperature orally twice daily for the next 21 days (or 21 days past their last possible exposure in an Ebola-endemic country). Beginning in December 2014, a pre-paid cellular telephone was provided, greatly aiding in establishing contact. Customs and Border Patrol (CBP) also screened all travelers for travel to one of the affected countries to identify instances of broken travel such as when someone travels from one of the Ebola affected countries to a second country, then travels by land to a third country before flying to the United States. CDC risk classification and contact information was immediately forwarded electronically to the state/local health department of the traveler’s ultimate destination including the Minnesota Department of Health (MDH) [4, 7]. MDH staff then began active monitoring of the traveler to ensure those with EVD symptoms could receive health care, if needed, in a coordinated fashion and that health care providers were prepared to safely provide care.

Prior to 2014, there has not been a prior similar attempt in the United States to monitor persons upon arrival based exclusively on travel from another country impacted by an infectious disease. Furthermore, the immediate health needs of persons arriving from Ebola-affected West African countries independent of possible EVD were largely unknown when the monitoring program began. Minnesota is the state with the largest Liberian population outside of Liberia [8]. We describe our experience establishing this monitoring program and describe the health needs of monitored travelers from Guinea, Liberia, and Sierra Leone.

## Methods

### Ethics Statement

This project was reviewed by CDC for human subject protection and determined to be non-research. [9]

### Public Health Infrastructure in Minnesota

In Minnesota, powers and duties of public health reside with the MDH Commissioner of Health, in contrast to many other U.S. states where such powers reside with local jurisdictions such as city or county health authorities. Clinical care is not directly provided by MDH or other state human service agencies; however, MDH and clinicians have a long history of collaborative approaches to managing communicable diseases. Testing for Ebola virus via reverse transcription-polymerase chain reaction (RT-PCR) was available to clinicians through the MDH Public Health Laboratory after consultation with MDH epidemiology and clinical staff.

### Monitoring Program

MDH began receiving names and contact information collected by U.S. CBP and CDC staff on October 21, 2014 via encrypted electronic communication. Traveler information was also received from other U.S. states if the traveler informed that state they intended to travel to Minnesota during the 21-day monitoring period. Monitoring staff from MDH, with the assistance of two county health agencies, telephoned the traveler and performed an additional risk exposure and health assessment within 24 hours of receipt of the traveler information. The monitor would reaffirm CDC-provided information on EVD symptoms using a list of symptoms provided by CDC as well as a general query of any illnesses or symptoms, and instruct them to contact MDH immediately (24 hours/day, 7 days/week) if they experienced any of those symptoms. For persons who were deemed “low (but not zero)” risk, as defined by CDC guidance (i.e. no known exposures) [4], daily calls with the monitor occurred where the traveler would provide twice daily temperatures obtained during the previous 24 hours and asked to report any symptoms potentially consistent with EVD (elevated body temperature or subjective fever or symptoms, including severe headache, fatigue, muscle pain, vomiting, diarrhea, abdominal pain, or unexplained hemorrhage). Monitored persons with “some” risk (i.e. being in close contact with a person with EVD while wearing appropriate personal protective equipment) underwent direct active monitoring involving daily in-person visits or video-teleconference conversation between the monitor and traveler in order to directly observe the taking of temperatures and reporting symptoms. All monitoring continued for 21 days after the traveler had left Guinea, Liberia, or Sierra Leone [10].

An MDH Ebola Clinical Team (ECT) was established, initially comprised of two MDH physicians rotating call weekly. This team was expanded after the first 3 months to include four additional doctorate-level epidemiologists (one Ph.D. epidemiologist and three veterinarians) with extensive human infectious disease experience. When a traveler reported symptoms or an oral temperature ≥100.4° F at any time during their 21-day monitoring period, an ECT member would speak to the traveler and perform a general open-ended clinical assessment by way of discussion by telephone or via video-chat. Based on the assessment the ECT member would recommend 1) continued clinical monitoring while at home with frequent telephone follow-up by the ECT member, 2) outpatient clinical evaluation at an outpatient site agreed upon by all parties, or 3) inpatient clinical evaluation. Four Minnesota Ebola Assessment and Treatment Units (EATUs) within hospitals with various overlapping capacities were established for the in-person clinical evaluation: two with capacity to care for adults, pediatric patients, and pregnant women; one to care for adults only; and one to care for pediatric patients only. The ECT member would contact an outpatient clinic or an EATU facility and direct the traveler there, taking into account the facility staff comfort level in providing anticipated care as well as traveler’s age, location, wishes, symptoms, and/or other health conditions (e.g. pregnancy status). Travel distance was estimated using city of residence as provided by the traveler to CBP upon entry to the United States. The distance from this location to the closest EATU was assessed and the shortest driving distance by car was selected for each location. The ECT member would notify the facility’s designated contact person about the ill traveler and, if requested, provide on-site consultation to healthcare providers if needed. ECT members assessed and approved testing for Ebola virus infection at MDH.

### Data Management

Date were collected and analyzed from the start of the program until May 15, 2015 since Liberia was declared Ebola-free on May 9, 2015 and the vast majority of travelers to Minnesota were from Liberia. All traveler data were stored in a single Microsoft 2013 Excel spreadsheet and managed by a single ECT member on a secure server. Information on the country of passport the traveler used at entry was collected by CBP. Calls to the ECT were logged with basic information about the traveler, symptoms, and clinical outcomes. Clinical information collected by MDH was based on report from the clinicians providing care when an in-person evaluation occurred.

## Results

**Fig 1:**
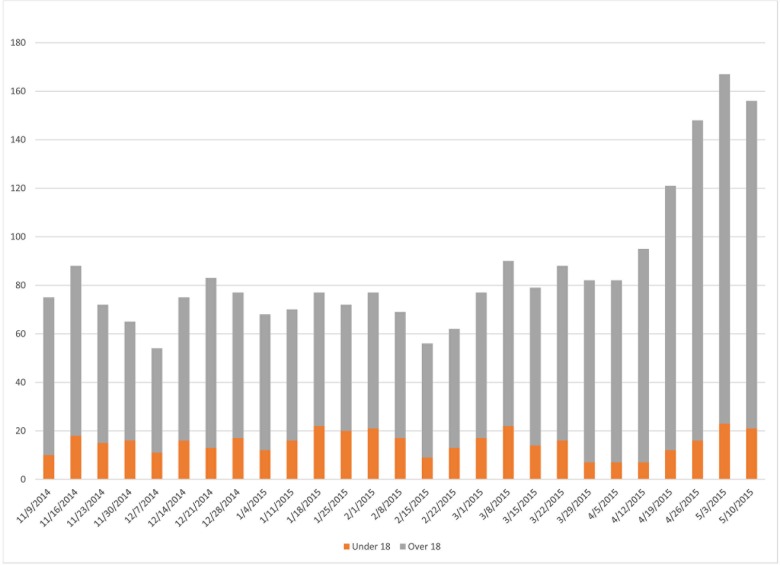
Weekly Numbers of Monitored Travelers by Age Category.

During October 21, 2014–May 15, 2015, a total of 783 travelers were monitored. The median number of monitored travelers each week was 59 with a range of 45–143 (Fig 1). Starting the week of April 19, 2015, there was an increase in the number of travelers to Minnesota (Fig 1).

The median age was 35 years, with 136 (17%) travelers aged <18 years. Thirteen (5%) of 248 females of reproductive age reported they were pregnant (Table 1). Travel from Liberia occurred in 729 (93%), whereas 30 (4%) traveled from Sierra Leone and 24 (3%) from Guinea. The majority of travelers, 478 (66%), traveled using a non-U. S. passport. Liberia was the most common country of issuance and accounted for 442 (61%) of all travelers. Seven hundred seventy-two (99%) travelers were considered “low (but not zero)” risk; 11 (1%) travelers were considered to be “some” risk; and none were considered “high” risk (i.e. direct contact with a person with EVD or their body fluids without appropriate personal protective equipment). The majority of travelers (710 [92%]) planned to stay in the 7-county Minneapolis-St. Paul metropolitan area and 724 (94%) stayed within a 30-mile driving distance from one of the EATUs (Table 1).

**Table 1:** Demographics of Monitored Travelers—Minnesota, October 21, 2014–May 15, 2015.

	All Monitored Travelers (% of all travelers)	Monitored Travelers Requiring Ebola Clinical Team Evaluation (% of travelers requiring Ebola Clinical Team Evaluation)
	N = 783*	N = 43
Age (years)	(n = 780)	(n = 43)
Median age	35 years	33 years
Age <5 years	44 (5%)	5 (12%)
Age 5–17 years	92 (12%)	5 (12%)
Age 18–40 years	348 (44%)	15 (35%)
Age >40 years	299 (38%)	18 (42%)
Female sex	395 (51%)	23 (54%)
Pregnant (n = 256^¶^)	13 (5%)	3 (1%)
Country where travel began	(n = 783)	(n = 43)
Liberia	729 (93%)	39 (91%)
Sierra Leone	30 (4%)	3 (7%)
Guinea	24 (3%)	1 (2%)
Exposure risk	(n = 783)	(n = 43)
High	0 (0%)	0 (0%)
Some	11 (1%)	3 (7%)
Low (but not zero)	772 (99%)	40 (93%)
Passport	(n = 720)	(n = 40)
Liberia	442(61%)	22 (55%)
United States	242 (34%)	17 (43%)
Sierra Leone	16 (2%)	1 (3%)
Guinea	11 (2%)	0 (0%)
India	4 (1%)	0 (0%)
Lebanon	2 (0%)	0 (0%)
France	1 (0%)	0 (0%)
Spain	1 (0%)	0 (0%)
United Kingdom	1 (0%)	0 (0%)
Location during monitoring	(n = 771)	(n = 42)
7-county Minneapolis-St. Paul metropolitan area^§^	710 (92%)	38 (93%)
Within 30 mile drive of EATU	724 (94%)	38 (93%)

* Total number of monitored travelers was 783. Different denominators noted when data elements missing.

^¶^ Women of reproductive age (15–49) per World Health Organization definition.

^§^ The statistical metropolitan area of Minneapolis-St. Paul includes the 7 counties of Anoka, Carver, Dakota, Hennepin, Ramsey, Scott, and Washington with a total population of 2,952,932 per 2013 U.S. Census. The population of the state of Minnesota is 5,420,380 per 2013 U.S. Census.

Of all travelers, 43 (5%) of 783 reported any signs or symptoms, of which 29 (67%) had a symptom consistent with EVD [11], 7 (16%) were admitted to EATUs, and 2 (5%) were tested for Ebola virus infection at MDH (Table 2). Travelers with symptoms were very similar in age, country of exposure, country of passport, and location of their planned stay compared with travelers who did not develop symptoms (Table 1). The most common symptom was self-reported fever, followed by abdominal pain, upper respiratory symptoms, and headache (Table 2).

**Table 2:** Clinical symptoms, evaluation, and diagnosis of monitored travelers requiring Clinical Ebola Team—Minnesota, October 21, 2014–May 15, 2015.

Signs and Symptoms	N = 43
Any symptom consistent with Ebola disease	29 (67%)
Temperature ≥100.4 F, or subjective fever	20 (47%)
Abdominal pain	12 (28%)
Cough, sore throat, and/or rhinorrhea	8 (19%)
Headache	6 (14%)
Myalgias	4 (9%)
Diarrhea	4 (9%)
Vomiting	3 (7%)
Unexpected bleeding	3 (7%)
Additional signs or symptoms*	15 (35%)
Ebola PCR performed	2 (5%)
Diagnoses by days from arrival to symptom onset^¶^	
Symptom onset prior to travel but condition not diagnosed until after arrival (n = 16)	Fibroids, bone mass, abdominal tumor, irritable bowel syndrome, dementia, developmental delay, atrial fibrillation, orthopedic fracture, pregnancy/labor, tubo-ovarian abscess, urinary outflow tract obstruction
≤7days (n = 9)	Upper respiratory tract infection, malaria, congestive heart failure, menstrual cramps, enterovirus, gastroenteritis, asthma exacerbation, atrial fibrillation, Gram-negative prostatitis
8–14 days (n = 11)	Upper respiratory tract infection, probable norovirus, panic attack, pre-term labor, epistaxis, respiratory syncytial virus
15–21 days (n = 7)	Upper respiratory tract infection, pre-term labor, probable norovirus
Highest level of care delivered	N = 43
Ebola Clinical Team via telephone/video call	15 (35%)
Outpatient clinical evaluation (including ED)	15 (35%)
Hospitalized	13 (30%)

* Additional signs and symptoms may have accompanied other signs or symptoms and included: hypertension (3), shortness of breath (2), elevated liver enzymes (2), nausea (1), thrombocytopenia (1), dehydration (1), bone pain (1), confusion (1), developmental delay (1), lower extremity edema (1), and chest pain (1).

^¶^ Ill travelers might have had multiple diagnoses.

Among the 43 travelers with symptom(s) evaluated by the ECT over the telephone or an in-person healthcare evaluation, 16 (37%) reported the symptoms (either intermittent or continuous) began prior to travel; chronic health conditions including tumors/cancer, pregnancy, and orthopedic conditions were most common (Table 2). Among those who had symptoms with onset after arrival, infectious causes were more common compared with non-infectious causes. With the exception of malaria, all infections diagnosed could have been acquired domestically in Minnesota or internationally (Table 2).

### Illustrative cases

A 32 year-old female “low (but not zero)” risk traveler and resident of Liberia thought she was 7 months pregnant with no prior prenatal care reported abdominal discomfort. ECT members secured an obstetric clinic visit. At that visit, the pregnancy was dated to be 33 weeks gestation and signs of pre-eclampsia were identified. She was afebrile and had no other symptoms consistent with EVD. She was admitted from clinic to a normal prenatal hospital bed within a health care facility with an EATU with plans to transfer her to the EVD isolation unit within the same building if any symptoms consistent with EVD developed in absence of another identifiable cause. No Ebola test was performed. Due to maternal and fetal indications, a Cesarean section was performed on hospital day 2. At 33 5/7 weeks gestation, a healthy infant was delivered and was transferred to the newborn intensive care unit. ECT members monitored the infant and mother by contacting members of each clinical team daily until the completion of the traveler’s 21 day EVD monitoring period. The remainder of the mother and infant’s hospitalizations were uneventful other than a single maternal temperature 100.4° F post-delivery which was attributed to postpartum breast engorgement.

A 2 year-old male was born in Minnesota within 2 days of his Liberian parents’ return from Liberia (after 5 months in Liberia) and was considered "low, but not zero risk" (as were his parents, who were also being monitored). The child was discharged to home 2 days after birth when he developed a low grade fever (unmeasured by parents) and diarrhea. Despite the travel monitoring guidance to call public health prior to going to any clinical setting, the family transported the mildly ill child to a local non-EATU hospital. The child was then transported to a children’s EATU hospital by ambulance. The child was evaluated in the emergency department (ED). The fever and diarrhea symptoms increased while in the ED prompting admission to the dedicated isolation room within the pediatric intensive care unit, and testing for Ebola and other infectious diseases. The child’s serum specimen was negative for Ebola virus on repeat RT-PCR testing and positive for Enterovirus by PCR. One challenge was how to handle parent presence when Ebola was suspected. In this case, the mother was allowed to stay in the EATU hospital room with the patient.

A 77 year-old male from Liberia who had “low (but not zero)” risk presented with 4 days of bloody urine to a non-EATU healthcare facility emergency department which notified MDH on monitoring day 1. He had a past history of chronic prostatic disease and similar episodes. The traveler had no fever or gastrointestinal symptoms. After discussions with the ECT he was admitted to that hospital for diagnosis and management of hematuria. Since his symptoms were inconsistent with EVD, routine infection control procedures were followed without additional personal protective equipment worn by staff. No Ebola test was performed. He subsequently experienced a temperature of 100.5° F while receiving a blood transfusion and care was continued at that facility after consultation with an ECT member. His hospital course was complicated by a urinary tract infection that was thought secondary to his prostatic obstruction. He underwent a radical prostectomy while hospitalized and was subsequently discharged home.

A 63 year-old male “low (but not zero)” risk traveler from Liberia was contacted within 24 hours of his arrival in Minnesota. On this initial call, he described active vomiting, abdominal pain, and feeling feverish. Emergency medical services were called by the ECT. Upon evaluation at the EATU he was noted to be mildly confused with rhinorrhea, cough, and myalgia and an ulcer on his shin which he reported had been there for some time. He had been to a funeral in Liberia 4 months prior to travel to Minnesota. A household member in Liberia had vomiting at the time of his departure; the patient was unaware of this person’s current health status. The traveler’s platelet count, liver enzymes, renal function, and glucose were all normal. Smear and PCR for malaria were negative. He was started on empiric ceftriaxone and metronidazole. Blood for Ebola PCR testing was collected within minutes of his arrival at the EATU. A preliminary negative Ebola PCR test result was reported to the EATU within 4 hours of specimen collection. Over the following 48 hours, daily Ebola virus disease RT-PCR test results were negative and he had substantial improvement in his abdominal pain and vomiting. He was discharged to home on hospital day 3 with home healthcare support with a diagnosis of acute gastroenteritis. He was readmitted to the same EATU 4 days later out of concern that he was unable to perform activities of daily living following home healthcare visits. An evaluation revealed signs of mild dementia. He agreed to nursing home placement but was not able to be transferred until monitoring day 17. Subsequent monitoring was completed in coordination with the nursing home staff; he did not further develop further EVD-like signs or symptoms.

## Discussion

Monitoring of travelers returning from Sierra Leone, Guinea, and Liberia during the West Africa Ebola outbreak was begun by CBP and CDC to minimize the consequences of further international spread of Ebola into the United States [4]. The program continues to screen travelers at many points prior to travel, during travel, and upon arrival at their final destination within the United States. In addition to the primary goal of preventing international spread of EVD, MDH staff was instrumental in ensuring safe, expedited access to healthcare for travelers needing urgent, non-Ebola related care during their 21-day observation period. This was exemplified by two instances of malaria with severe outcomes (preventable ICU stay and death) resulting from a delay in care that occurred prior to the initiation of the monitoring program in Minnesota.

In Minnesota, the majority of monitored travelers were from Liberia. Many were traveling using non-U.S. passports suggesting that they were visitors or temporary residents in Minnesota. This was likely different than other U.S. states where many travelers were U.S. government employees or aid workers in the affected countries who resided in the United States [12, 13]. MDH received the seventh largest number of travelers in the United States but very few were government or aid workers.

The infectious causes of acute symptoms we identified including malaria, acute diarrheal disease, and respiratory viral infections and were consistent with past assessments of illness following travel to these countries and persons seeking care in-country at Ebola treatment units [14, 15]. Among our travelers, 17% were <18 years of age, and 5% of 248 females of reproductive age were pregnant, requiring unique processes to ensure appropriate care. Many travelers also volunteered to monitor staff that they traveled to Minnesota for the specific purpose of seeking treatment for chronic diseases. Many had no existing connection to the healthcare system. Care of chronic conditions frequently was delivered by EATU facilities as other facilities felt ill-prepared to meet the needs of an ill monitored traveler if they also developed a symptom consistent with EVD. On several occasions, symptoms that developed during their monitoring period were consistent with known chronic health conditions (i.e. abdominal tumor or fibroids causing abdominal pain). When a patient remained at a non-EATU facility, extensive effort by the ECT and staff at both the non-EATU and the EATU was required to develop transfer plans if the patient’s clinical condition changed. There were instances where ECT staff needed to convince healthcare facilities to provide urgent medical care for chronic health conditions that could not be delayed until the completion of the 21 day monitoring period when the risk of delayed care far exceeded the risk of developing EVD.

Only two monitored patients were tested for Ebola virus infection and both had negative results; both patients had been classified as “low (but not zero)” risk. Repeat clinical reassessment after a few hours of observation was a common approach used by both ECT and EATU staff. In multiple instances, symptoms improved or evolved making other diagnoses much more obvious.

There was variability in the implementation of monitoring programs in various jurisdictions across the United States [16]. However, given that no additional cases of EVD were identified following implementation of the Ebola Monitoring Program underscores the success of the screening prior to travel, public health messaging in affected countries, and the type of exposure required to lead to transmission of EVD. Twice daily monitoring of fever and reporting to monitors was emphasized as a key screening tool for travelers. While not all cases of EVD have fever [17], this has been consistently used by military and non-military monitoring following travel to affected countries [12, 13]. In Minnesota, the regular contact between monitoring staff and traveler to discuss all symptoms and overall health status was the most important part of the monitoring, leading to reduced false alarms of possible EVD; this success, however, was resource-intensive.

The traveler monitoring program presented many unanticipated challenges beyond screening for EVD. Public health staff were put into the unique roles of serving as social workers or care triage agents as has been described elsewhere [18, 19]. They were often compelled to directly intervene in the medical care of persons, directing the monitored travelers on where to go for assessment and treatment, helping to arrange for transport, and making care decisions collaboratively with care providers. Extensive communication with the EATU was required each time a traveler was referred for evaluation to assure proper infection control measures were in place and specialists ready upon traveler arrival. In Minnesota, a large proportion of the conditions were chronic non-infectious conditions that predated the travelers’ arrival and had, not infrequently, been previously untreated. This program continues to require considerable resources with 29,689 travelers having been screened as of September 17, 2015 and no cases or subsequent cases of EVD identified through this program [20]. Before similar monitoring programs are considered in the future, careful thought must be given to the necessary resources and the impact on affected populations, public health, and the healthcare system.
